# Developing mental health services during and in the aftermath of the Ebola virus disease outbreak in armed conflict settings: a scoping review

**DOI:** 10.1186/s12992-022-00862-0

**Published:** 2022-07-14

**Authors:** Bives Mutume Nzanzu Vivalya, Martial Mumbere Vagheni, Germain Manzekele Bin Kitoko, Jeremie Muhindo Vutegha, Augustin Kensale Kalume, Astride Lina Piripiri, Yvonne Duagani Masika, Jean-Bosco Kahindo Mbeva

**Affiliations:** 1 Department of Psychiatry, Kampala International University Western Campus, P.O BOX 71 Bushenyi, Uganda; 2 Department of Internal Medicine, Masereka General Referral Hospital, Goma, North-Kivu Democratic Republic of the Congo; 3 Department of Psychiatry, University of Kinshasa, Kinshasa, Democratic Republic of Congo; 4 Division provinciale de la Sante, Goma, North-Kivu Democratic Republic of Congo; 5 Kinshasa School of Public Health, Faculty of Medicine University of Kinshasa, Kinshasa, Democratic Republic of Congo; 6 Faculté de Psychologie et des Sciences de l’Education, Université de Kinshasa, Kinshasa, République Démocratique du Congo; 7 Department of Public Health, Official University of Ruwenzori, Goma, North-Kivu Democratic Republic of the Congo

**Keywords:** Mental health, Mental health services, Ebola, Democratic Republic of Congo, Scoping review, Armed conflict

## Abstract

**Background:**

Mental health is mostly affected by numerous socioeconomic factors that need to be addressed through comprehensive strategies. The aftermath of armed conflict and natural disasters such as Ebola disease virus (EVD) outbreaks is frequently associated with poor access to mental healthcare. To design the basis of improving mental health services via the integration of mental health into primary health care in the Democratic Republic of Congo (DRC), we conducted a scoping review of available literature regarding mental illness in armed conflict and EVD outbreak settings.

**Methods:**

This scoping review of studies conducted in armed conflict and EVD outbreak of DRC settings synthesize the findings and suggestions related to improve the provision of mental health services. We sued the extension of Preferred Reporting Items for Systematic Reviews and Meta-Analyses to scoping studies. A mapping of evidence related to mental disorders in the eastern part of DRC from studies identified through searches of electronic databases (MEDLINE, Scopus, Psych Info, Google Scholar, and CINAHL). Screening and extraction of data were conducted by two reviewers independently.

**Results:**

This review identified seven papers and described the findings in a narrative approach. It reveals that the burden of mental illness is consistent, although mental healthcare is not integrated into primary health care. Access to mental healthcare requires the involvement of affected communities in their problem-solving process. This review highlights the basis of the implementation of a comprehensive mental health care, through the application of mental health Gap Action Program (mhGAP) at community level. Lastly, it calls for further implementation research perspectives on the integration of mental healthcare into the health system of areas affecting by civil instability and natural disasters.

**Conclusion:**

This paper acknowledges poor implementation of community mental health services into primary health care in regions affected by armed conflict and natural disasters. All relevant stakeholders involved in the provision of mental health services should need to rethink to implementation of mhGAP into the emergency response against outbreaks and natural disasters.

## Introduction

Mental health is usually affected by a range of socioeconomic factors that need to be addressed through comprehensive strategies [1]. In armed conflict and Ebola disease virus (EVD) outbreak contexts, individuals are at higher risk of experiencing mental health problems such depression, anxiety, substance abuse and post-traumatic stress disorders [2]. Mental disorders are major public health concerns in low-income countries including the Democratic Republic of Congo (DRC) [3]. To efficiently reduce the burden of mental disorders in these countries, the World Health Organization (WHO) emphasizes the provision of mental health services into primary health care (PHC) via the implementation of mental health Gap Action program (mhGAP) [4]. Hitherto, important inefficiencies remain unresolved in DRC which is characterized by a high concentration of trained health workers in big cities while leaving the countryside uncovered [5].

Public health emergencies are disproportionally linked to an increasing burden of mental illness among communities, especially those with existing psychological vulnerabilities [2]. Recent studies highlight an urgent need for the building of a strong mental health system using existing health ecosystem in conflict zone and outbreak settings.

Overtime, the integration of mental healthcare into PHC has been affected by lack of funds, scarcity of specialized workers, lack of mental health legislation, and impaired mental health information at different levels of the health system [6]. These challenges are more likely expressed during public health emergency, and affect the provision of short-time psychological aid to direct victims and survivors by the emergency response team [7].

To date, DRC lacks sufficient trained health workers able to implement mental healthcare services based on the recommendations of mhGAP in several provinces. Most of persons with mental disorders receive treatment mental health facilities which, not only belong to private sectors but also are equipped by non-specialists workers. Furthermore, poor access to mental health services in public facilities is due to low coverage of mental health providers by 100,000 population in DRC [8].

Therefore, to design the basis of improving mental health services via the integration of mental health into PHC in the eastern part of DRC, we conducted a scoping review of available literature conducted in this aforementioned region in order to better analyze the burden of mental health problems.

## Methods

The scoping review approach was performed to conduct this study, given that it is well-established as the first step in research evidence development [9]. We used the methodological framework described by the Joana Briggs Institute [10] and the extension of Preferred Reporting Items for Systematic Reviews and Meta-Analyses (PRISMA) to scoping reviews [11]. This review was neither published nor registered. It analyzes the available literature regarding the burden of mental disorders and the availability of mental healthcare services in conflict zone and EVD outbreak settings of DRC, in order to identify suggestions from these studies able to increase the implementation of the mhGAP to this specific setting. The topic does not follow the Population Intervention Comparison Outcome model, given that we focus on mapping the existing literature in areas where armed conflicts have spent more than two decades [12].

Literature search used a grey literature approach conducted by two independent experts (BMNV and MMV) who collected and extracted data using Medline, Scopus, Psych Info, Google Scholar, and CINAHL, using the following keywords: “mental illness in DRC”, mental illness in EVD outbreak settings”, “the collision of EVD outbreak and armed conflict”, “Mental illness during EVD outbreak and armed conflict in DRC”. Search results were uploaded into Endnote software; duplications were removed through the control quality analysis.

This scoping review of studies involved the screening of the abstract and full text of each article to check whether it included findings regarding the availability of mental healthcare services as well as the burden of mental disorders in the eastern part of DRC. Abstracts and full texts were analyzed for published studies including short communication, commentaries, original studies, and reviews. A pretested template was approved by all the authors regarding the focus of the study. We only included studies that including participants who were direct or indirect victims of armed conflict and EVD outbreaks.

A narrative synthesis and discussion of the findings were performed in order to achieve the objective of this study. We collected data on study design, content and scope of the study, the main findings, and the suggestive measures to improve the mental healthcare services in the Eastern DRC. Relevant studies were identified using citation mapping. We selected published and peer-reviewed articles from January 2013 to August 2021.

## Results

Grey literature searches yield a total of 49 records, among 11 duplicated papers that were removed from the database. From the 38 studies selected, the assessment of titles and abstracts set up 16 papers recruited. We included papers that summarize the implementation of mental healthcare, those that highlight the burden of mental illness, and those who suggested reforms for the provision of mental health services. The careful review of inclusion criteria and the revision of the full text bring out the sample of 6 studies that are included in this review. Ten articles were not included, given that they did not provide important information targeted by the objective of this review. In DRC, mental healthcare is characterized by a lack of infrastructure and trained mental workers [6]. To fight the paucity of mental health-related resources, the provision of health services at community level of DRC’s health system may increase the strategies targeting by mhGAP [13].

The first study included by this review concerned adults receiving healthcare at mental health units in Butembo city (North-Kivu province) and revealed that 60% of study participants reported lacking needed mental healthcare services prior to admissions. Also it found that predictors of affective and psychotic disorders were death of a loved one, history of sexual abuse, history of childhood trauma, and being kidnapping. This study suggested that both the relatives and community health workers should be involved in close monitoring of people with psychological distress during civil unrest and outbreaks [14]. The second study assessed 144 EVD survivors and found that a young age increases the risk of developing post-traumatic stress disorder. This study suggested that the public emergency response team against the EVD outbreak should promote the basic building of psychiatric services to sustain mental health among survivors of EVD outbreaks [15]. Thirdly, Duagani and colleagues found high scores of peritraumatic dissociation among participants with post-traumatic stress disorders while screening 120 individuals aged between 17 and 75 years old. In addition, being physically or sexually abused and low education level were more likely associated with dissociation during stressful events exposure than compared to witnesses and those with a higher level of education. This study suggested that the primary target population for prevention and early management should comprise individuals with high levels of peritraumatic dissociation, low levels of education, and women [16].

Fourthly, a study pre-testing the integration of mental health services in rural zone reported that the average utilization rate of primary health centres for mental health problems was 7 new cases per 1000 inhabitants per year. The majority of patients were treated on an outpatient basis. This study indicates that the success of integration mental health into PHC depends on the quality of existing health system and the involvement of and non-health actors, including community leaders. Furthermore, this study showed that the major problems in terms of access and use of basic care indicate that the successful integration of mental health depends on the involvement of health and non-health actors [6]. In these settings, armed conflicts and outbreaks were reported to be associated with severe mental illnesses among subjects victims of sexual violence [17]. Fifthly, Dossa and colleagues revealed that women who experienced sexual abuse associated with fistula lacked psychosocial support and are more likely exposed to mental health problems. This study shows that psychological and physical healthcare services are needed for women who experienced conflict-related disorders [18]. Lastly, Hecker and colleagues found that forcing army recruitment was associated with a high risk of mental illness what is more likely to be complicated by the poor provision of needed mental healthcare. Therefore, these authors suggested that a clear consideration could be emphasized on the combatant’s perception of committing violent acts [19].

Results of the available studies in DRC evidenced the mental health challenges and their contribution to the burden of mental illness among individuals living in a region affected by natural disaster/armed conflict settings. Also, they highlighted the need of involving mental health non-specialist and specialist in treating patients with mental health problems at daily or weekly basis [6].

## Discussion

This paper reviews the existing literature on burden attributed to mental health in the Eastern DRC and summarizes the suggestive means of amelioration of integration of mental health into PHC. Although developed countries have implemented guidelines to cope with the increasing health challenges during armed conflict or natural disasters, few efforts have been done in developing countries to cover the burden of psychological distress and mental disorder during pandemics [20].

We found that mental health services are not integrated into PHC across the eastern part of the country, despite the wide recognition of its contribution to the health system. In fact, there is less attention regarding the application of mental health legislation during public health emergencies. To date, less than 10% of individuals with mental illness have access to needed healthcare services in DRC [6]. Additionally, a recent study revealed high rates of relapse among adolescent patients living in these armed conflict and EVD outbreak settings in DRC [21]. In most cases, the majority of Congolese population travel a distance of more than 10 km before attending mental health facilities [22]. Mental health care is frequently provided at the health facility level, mainly for individuals with high educational and socioeconomic status [6].

There is strong evidence that outbreaks and armed conflict impeded the quality of life. A recent study demonstrated that 28.0% of the global population experienced depression; 26.9% of cases showed anxiety; 24.1% of cases presented the symptoms of post-traumatic stress disorder; and sleep-related problems were seen in 27.6% of cases during COVID-19 [23]. Mental health problems during outbreaks and armed conflict are not fully treated due to lack of health workers or stigma. There is an urgent need for a strong mental health system, centered on improving the provision of mental health services into PHC and communities. This may increase access to mental healthcare services to affected individuals. Wakida and colleagues suggested that the achievement of good outcomes during the management of mental illness requires adaptation of national guidelines regarding mental health into the local context of health care [24]. Therefore, the integration of mental healthcare into PHC remains the best alternative to the provision of mental healthcare at both community and health facilities levels.

The eastern part of DRC has a large range of risk factors that can result in an explosion of mental disorders. While mental health is not fully integrated into health care among people living in armed conflict and outbreaks, except for the survivors of gender-based violence; mental illness is a major concern of public health [25]. New policies based on the extension of emergency response to the existing health system are required regarding the promotion of guidelines on how to handle mental health challenges due to outbreaks. Amelioration of mental health services into PHC requires appropriate planning, continuous monitoring, and measurement of performance as well as their recruitment among and into the concerned communities [8]. Our analysis of existing evidence regarding mental illness in the DRC highlights the importance of the implementation of a new model of providing mental healthcare services.

### Improving mental health services during and in the aftermath of the Ebola virus disease outbreak in armed conflict settings

As a result of the analysis of available evidence; a model based on the implementation of mhGAP at community level of the health system is actually proposed by this review. This model should aim to ameliorate mental healthcare services via the community engagement [26]. Active encouragement toward the adoption and diffusion of the mhGAP services sets up a basis of providing mental health of quality across individual communities.

To ensure that access to mental health services has improved, social workers and community health workers should be skilled and supported to reach all households at least weekly for collecting and addressing mental health problems [4]. Also important skills are needed by the health zone monitors and workers to implement the mental healthcare services at community levels. Additionally, even if the mental health department is implemented at the Ministry of Health of DRC and has established the mental health legislation [6], its priority should ensure that the success of the mhGAP in complex humanitarian setting of DRC helps to achieve universal health coverage in regions affected by armed conflict and health emergencies regarding the mental health. Mental health promotion and the establishment of a close relationship between modern mental healthcares with community, traditional and religious healers could improve the mental health of Congolese.

First, raising the awareness of mhGAP programs during outbreaks and armed conflict needs important reforms of mental health legislation [27]. Secondly, the public emergency response against the tenth and twelfth EVD outbreaks in DRC demonstrated an important delay to overcome community resistance, triggered by psychological and mental background of the affected community [3], despite the involvement of experts to cover the gap of mental health workers in the local context [2]. Prioritization of community mental health support for direct and indirect victims of natural disasters highlights the implementation of mental wellness check-ups of vulnerable individuals and continuous monitoring [4]. This could be shifted from the short-term period, commonly used to, long-term mental health programs. Therefore, these trained health workers should be prioritized as part of the mental health task force for the implementation of community mental health into the health system in the aftermath of outbreaks.

Thirdly, the implementation of mental health services at PHC requires the development of standardized approaches to use in outbreak and conflict zones settings. Furthermore, this implementation highlights the change of support provided to PHC workers and health communication regarding community mental health and psychosocial support. Mental health could be included in the national country’s health communication systems, and a need for developing specific screening psychological tools useful by health care providers and policymakers at the provincial, operational, and community levels to strengthen mental capacity building.

### Further research perspectives

To ensure the integration of the mental health model into PHC in the aftermath of armed conflict and outbreaks; there is a need of implementation research study related to the mhGAP in three provinces of eastern DRC, namely South Kivu, North Kivu, and Ituri. This research will aim to identify the barriers and facilitators to scaling up mhGAP interventions and the integration of mental health services into PHC. This study will target to improve the uptake of the findings research for effective development of new policy in DRC. Furthermore, this study will address the following objectives: i) to identify operational strategies, implementation challenges, and gaps of the mhGAP interventions in conflict settings, and propose solutions with a potential influence of policies and practices of mental healthcare services in the three above-mentioned provinces; ii) to explore the factors influencing the proposed model to contribute to the promotion of mental health and well-being of individuals living in armed conflict concerned with EVD outbreaks; iii) to identify lessons about the implementation of a mhGAP regarding the prevention and management of mental illness as well as the promotion of mental health; iv) to propose feasible solutions able to determine the sustainability of the community mental health and psychosocial support model proposed by this paper.

A multilevel strategy will be performed for an in-depth understanding of the process of integration of mental health services into PHC. A mixed approach using qualitative and quantitative will be used to collect data. Measurement will concern the evolution of common mental illnesses over time; the access to mental health facilities, as well as the involvement of all the relevant stakeholders in the provision of mental healthcare into PHC. Desk review, in-depth interviews and focus group discussions associated with consultation and brainstorming will be used to collect data that will have the potential to influence the control and promotion of mental health and well-being at the community level. Recommendations of this study will offer insight to all the relevant stakeholders including the Ministry of Health on the barriers and facilitators to scaling up mhGAP interventions and the integration of mental health services into PHC. However, these recommendations have to be read in DRC context.

## Conclusion

We have tried to provide insight into a safe space where communities can access mental health services during and in the aftermath of public health emergencies and armed conflict. The review of literature existing in DRC revealed that EVD outbreaks and armed conflict are important factors of mental disorders in both survivors and caregivers. All relevant stakeholders involved in the provision of mental health services should need to rethink to implementation of mhGAP into the emergency response against outbreaks and natural disasters.

## Data Availability

Not applicable.

## Abbreviations

COVID-19: Coronavirus disease pandemic
DRC: Democratic Republic of Congo
EVD: Ebola virus disease
mhGAP: Mental health Gap Action program
PHC: Primary Health Care
PRISMA: Preferred Reporting Items for Systematic Reviews and Meta-Analyses
WHO: World Health Organization.
